# Effects of hyperglycemia on the progression of tumor diseases

**DOI:** 10.1186/s13046-019-1309-6

**Published:** 2019-07-23

**Authors:** Wenjie Li, Xuehui Zhang, Hui Sang, Ying Zhou, Chunyu Shang, Yongqing Wang, Hong Zhu

**Affiliations:** 1 Department of Gastroenterology, The First Affiliated Hospital of Nanjing Medical University, 300 Guangzhou Road, Nanjing, 210029 China; 2 Department of Pharmacy, The Affiliated Jiangsu Shengze Hospital of Nanjing Medical University, Suzhou, 215228 China; 3 Research Division of Clinical Pharmacology, The First Affiliated Hospital of Nanjing Medical University, 300 Guangzhou Road, Nanjing, 210029 China

**Keywords:** Hyperglycemia, Tumor cells, Correlation, Mechanism, Progress

## Abstract

Malignant tumors are often multifactorial. Epidemiological studies have shown that hyperglycemia raises the prevalence and mortality of certain malignancies, like breast, liver, bladder, pancreatic, colorectal, endometrial cancers. Hyperglycemia can promote the proliferation, invasion and migration, induce the apoptotic resistance and enhance the chemoresistance of tumor cells. This review focuses on the new findings in the relationship between hyperglycemia and tumor development.

## Background

Recent studies have recognized hyperglycemia as a factor for cancer development in patients with diabetes. (The diabetes mentioned in this article is mainly type 2 diabetes). Hyperglycemia increases the prevalence and mortality (either short- or long-term) of many malignancies [1–5]. According to the WHO data, the number of patients with diabetes will increase from 382 million in 2015 to 592 million in 2035 [6]. Patients with diabetes face an increased risk of developing cancers, mainly including breast, liver, bladder, pancreatic, colorectal, endometrial cancers [7] (Table.1). This risk may arouse from special diabetic pathology, such as hyperglycemia, hyperinsulinemia, insulin resistance, distorted insulin-like growth factor-1 (IGF-1) pathway, oxidative stress, enhanced inflammatory processes, and aberrant sex hormone production [8, 9]. Studies have shown that hyperglycemia is one of the key factors in the hypothesis that diabetes is at increased risk of cancer [10–12]. Warburg O. first proposed that elevated blood glucose was associated with tumorigenesis [13]. Since then, many researchers have found that hyperglycemia can promote tumor development [1–3].

**Table 1:** Diabetes is a risk factor for cancer (summary of meta-analyses)

		Case-control studies		Prospective cohort studies	
Authors	Tumor	n	RR (95% CI)	n	RR (95% CI)
Wolf et al.	Breast	4	1.1 (1.0–1.3)	6	1.3 (1.2–1.3)
El-Serag et al.	HCC	13	2.5 (1.9–3.2)	12	2.5 (1.9–3.2)
Huxley et al.	Pancreatic	17	1.9 (1.5–2.5)	19	1.7 (1.6–1.9)
Larsson et al.	Colorectal	6	1.4 (1.2–1.5)	9	1.3 (1.2–1.4)
Larsson et al.	Bladder	7	1.4 (1.0–1.8)	3	1.4 (1.2–1.7)
Friberg et al.	Endometrium	13	2.2 (1.8–2.7)	3	1.6 (1.2–2.2)

*CI* confidence interval, *RR* pooled relative risk

The glucose metabolism in tumor cells is characterized by the “Warburg” effect. Under aerobic or anoxic conditions, the cells initiate glycolysis to convert glucose into lactic acid, a process in which energy is produced [14]. Because of the deficiency of adenosine triphosphate (ATP) produced by glycolysis, the tumor cells increase the intake of glucose to boost energy-providing glycolysis. High glucose level supports tumor progression through a variety of mechanisms, including promoting tumor cell proliferation, invasion and migration and inducing apoptotic resistance and chemoresistance. However, more mechanisms may also be involved. This review aims to explore the mechanisms engaging hyperglycemia with tumor cell behavior, which we hope to benefit the treatment for cancer patients with diabetes.

### Effect of hyperglycemia on tumor cell proliferation

Joshi et al. [15] pointed out that hyperglycemia could provide nutrients for the rapid proliferation of malignant tumor cells, thereby accelerating the process of tumor cells. Hou et al. [16] reported that high-concentration glucose (25 mM) significantly increased the proliferation of breast cancer cells (such as MDAMB231) compared to low-concentration glucose (5 mM). The mechanism may be that epidermal growth factor receptor (EGFR) is activated by guanosine triphosphatases (GTPases) Rac1 and Cdc42 to accelerate cell cycle progression and promote breast cancer cell proliferation. Han et al. [17] revealed that the proliferation of pancreatic cancer cells (such as BxPC-3 and Panc-1 cells) was affected by glucose concentration: high glucose (25, 50 mM) significantly increased pancreatic cancer cell proliferation compared to low glucose (5.5 mM). High glucose-induced epidermal growth factor (EGF) expression and EGFR transactivation may increase pancreatic cancer cell proliferation.

Long-term hyperglycemia leads to the production of a wide range of pro-inflammatory factors, such as interleukin-6 (IL-6), tumor necrosis factor-α (TNF-α), cyclooxygenase-2 (COX-2). These factors may be closely related to the development of tumors. Pothiwala et al. [18] pointed out that cytokines such as IL-6, TNF-α and COX-2 could stimulate oncogene expression, regulate cell cycle, promote tumor cell proliferation, inhibit apoptosis, and even induce epithelial-to-mesenchymal transition (EMT). EMT is widely recognized in cancer progression by enhancing cell invasion and anti-apoptosis [19, 20]. In EMT, polarized epithelial cells interact with the basement membrane through their basal surface and biochemically differentiate into interstitial phenotypes, a process through which invasive and anti-apoptotic properties are endowed and extracellular matrix generated.

Flores et al. [21] showed that high glucose (30 mM) increased the proliferation of breast cancer cells (MDA-MB-231) compared with low glucose (5.6 mM), and increased insulin further enhanced the proliferative effect of high glucose. High glucose (or high glucose and insulin)-induced cell proliferation may be mediated, at least in part, by oxidative stress, in which plasminogen activation is regulated by reactive oxygen species (ROS) production. Li et al. [22] found that hyperglycemia could induce miR-301a expression in prostate cancer cells in rat models and that miR-301a expression could inhibit the expression of p21 and Smad4, thus promoting the cells cycle from G1 and S phase, tumor cell proliferation and xenograft growth in nude mice. p21 is a cyclin-dependent kinase (CDK) inhibitor that blocks the cell cycle from G1 to S phase [23], and Smad4 can also induce G1/S cell cycle arrest [24]. Other researchers also pointed out that miR-301a promoted human tumor progression [25, 26], confirming the finding of Li et al.

Wang et al. [27] found that hyperglycemia could induce angiogenesis and tumor growth through hypoxia-inducible factor-1/vascular endothelial growth factor-dependent (HIF-1/VEGF) pathway. The mechanism may be that hyperglycemia, by impairing the function of HIF-1 inhibitors, attenuating the resistance of HIF-1 inhibitors against tumor chemotherapy or radiotherapy, increases tumor microvascular formation and tumor growth. They also pointed out that the prognosis of patients with hyperglycemia treated with HIF-1 inhibitor might be worse than those with low blood glucose. Other investigators also found that hyperglycemia promoted the proliferation of malignant breast cancer epithelial cells by increasing leptin/insulin-like growth factor-1 receptor (IGF-1R) signaling and activating the Protein Kinase B/mechanistic target of rapamycin (AKT/mTOR) pathway [28].

### Effect of hyperglycemia on tumor cell invasion

Matrix metalloproteinase-2 (MMP-2), a member of the MMPs family, is involved in the breakdown of extracellular matrices, a process promoting tumor invasion [29]. Compared with cholangiocarcinoma cells cultured in low glucose, those cultured with high-concentration glucose showed stronger activation of signal transducer and activator of transcription3 (STAT3) and higher expression of MMP2 in the downstream of STAT3. Lowering blood glucose or using STAT3 inhibitors reduced the invasion of cholangiocarcinoma cells, so Saengboonmee et al. [30] pointed out that hyperglycemia might increase the invasive ability of biliary tumor cells by activating STAT3. Resveratrol could inhibit the proliferation and invasion of liver cancer cells by inhibiting the expression of STAT3 gene in high glucose environment [31].

Kang et al. [32] treated human lung epithelial cells (A549) with high-concentration glucose, finding that the expression of heme oxygenase-1 (HO-1) in cells increased. Moreover, cluster of differentiation 147 (CD147) and MMP-9, two strains of HO-l mediated protein associated with tumor cell invasion and metastasis, also showed increased expression that as a consequence enhanced tumor cell invasiveness. If HO-1 expression was silenced, high glucose-induced protein expression was reduced and tumor cell invasiveness attenuated. The mechanism may be that HO-1 expression is increased by hyperglycemia mediated by up-regulation of ROS or the TGF-β1/PI3K/Akt signaling pathway. In malignant tumors, such as lung cancer and bladder cancer, up-regulation of HO-1 is a factor for cancer poor prognosis [33, 34].

Alisson et al. [35] found that hyperglycemia (25 mM) induced TGF-β secretion in human lung cancer cell A549 compared with low blood glucose concentration group (5 mM). TGF-β is an important inducer of EMT and TGF-β signal conduction can lead to EMT [36] that enhances cell invasion and anti-apoptosis in cancer progression [19, 20]. Both Flores and Viedma have shown that high glucose can promote breast cancer cell invasion by inducing EMT [21, 37].

**Fig. 1:**
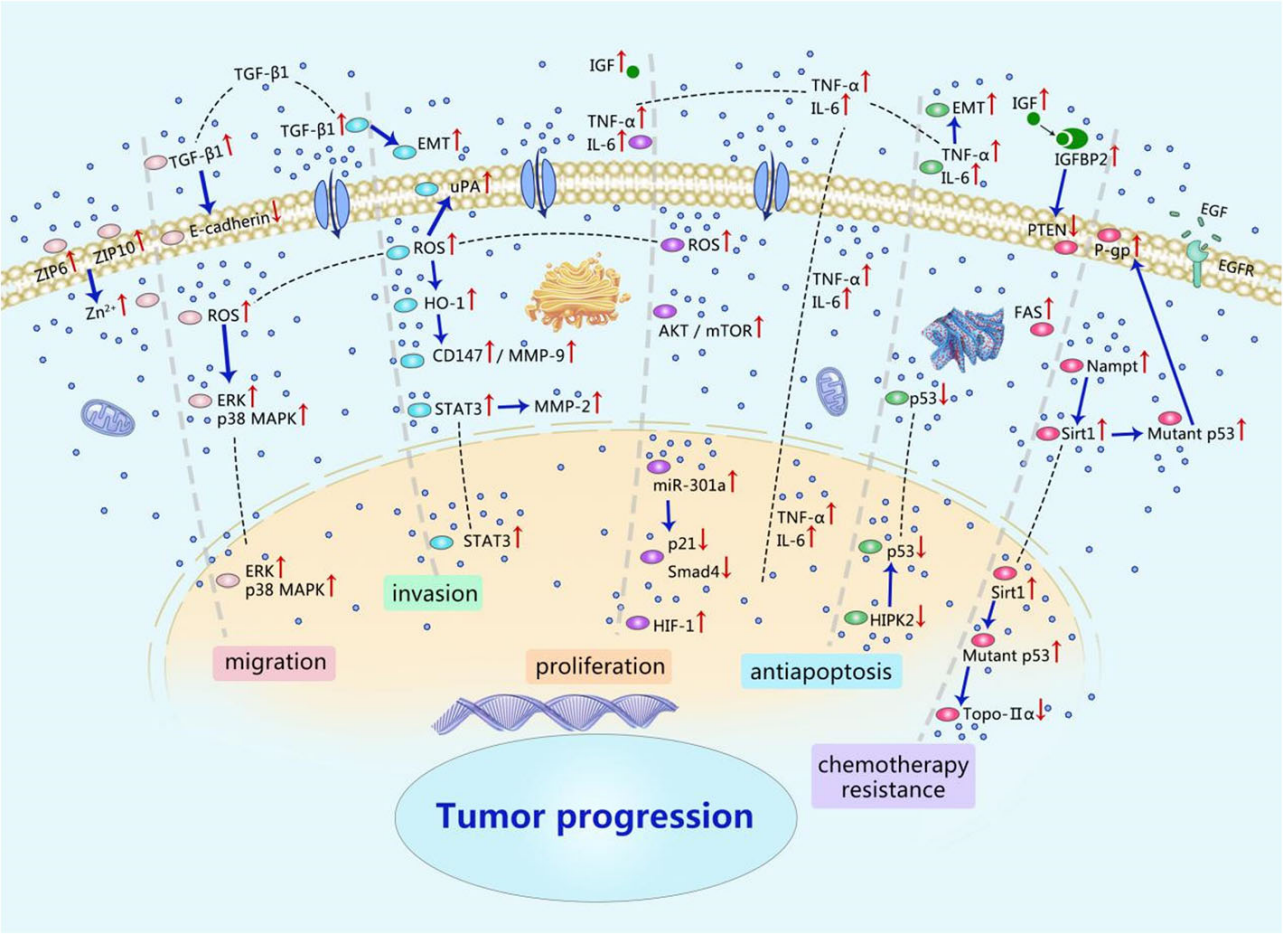
Mechanisms underlying hyperglycemia-promoted cancer progression

Sun et al. [38] demonstrated by transwell experiments that compared to those cultured in low glucose (5.56 mM), the breast cancer cells 7 cells (MCF-7) cultured in high glucose (25 mM) medium had stronger invasive ability, while the mRNA and protein expression of Glut1, MMP2 and MMP9 were significantly increased; besides, down-regulating Glut1 inhibited the invasion of MCF-7 cells and inhibited the expression of MMP2 and MMP9. Flores et al. [21] pointed out that hyperglycemia also increased the expression of serine protease urinary plasminogen activator (uPA) in tumor cells through ROS. uPA can proteolyze extracellular matrix components and the basement membrane around the primary tumor, thereby promoting tumor cell invasion. In summary, hyperglycemia can increase the expression of MMPs and uPA, the hydrolysis of extracellular matrix components, the invasion of tumor cells into adjacent normal tissues (Fig. 1).

### Effect of hyperglycemia on tumor cell migration

Li et al. [39, 40] reported that hyperglycemia could promote the migration and invasion of pancreatic cancer cells (such as BxPC-3 and Panc-1 cells). The possible mechanism is that hyperglycemia can increase the concentration of H_2_O_2_ by up-regulating the expression of manganese superoxide dismutase (SOD2), and then activate the extracellular signal-regulated kinase (ERK) and protein 38 mitogen-activated protein kinases (p38 MAPK) pathways. H_2_O_2_ is a key factor mediating hyperglycemia-induced pancreatic cancer cell migration and invasion. After the addition of SOD2 inhibitor and polyethylene glycol-conjugated catalase (PEG-CAT), the migration was effectively inhibited. In vitro and in vivo studies showed that H_2_O_2_ increased the invasive and migratory ability of pancreatic cancer cells, and their invasion and migration were terminated after being treated with PEG-CAT.

Rahn et al. [41] studied precancerous H6c7-kras pancreatic cells with epithelial features, finding that hyperglycemia activated TGF-β1 signaling by increasing TGF-β1 expression and secretion, leading to a decrease in the expression of downstream Smad-dependent E-cadherin, which was more likely to break away from the mass and invade surrounding tissues, thereby promoting tumor cell metastasis. Takatani et al. [42] found that MCF-7 cultured in high glucose (25 mM) showed increased motility compared those cultured with low glucose (5.5 mM). The difference may be achieved by Zn^2+^ transported by Zin transporter 6 (ZIP6) and Zin transporter 10 (ZIP10). Zn^2+^ plays a crucial role in glucose-induced cell migration. Lack of Zn^2+^ significantly weakens the migrative activity of breast cancer cells in hyperglycemic conditions.

Together, it can be seen that the hyperglycemia promotes the migration of tumor cells, and the migration of tumor cells determines the quality of life and survival time of patients with advanced cancer to some extent. In general, highly migrative tumor cells are always highly invasive. Therefore, in the hyperglycemic environment, invasion always synchronizes migration, both deciding the prognosis of cancer patients.

### Effect of hyperglycemia on tumor cell apoptotic resistance

p53 can suppress the canceration of cells and activate tumor cells’ response to anticancer drugs [43]. Garufi et al. [43] pointed out that hyperglycemia could inhibit p53 pro-apoptotic properties by reducing p53 phosphorylation of serine 46 (Ser46). Homeodomain-interacting protein kinase 2 (HIPK2) is a nuclear serine/threonine kinase that regulates the p53-dependent apoptotic pathway and tumor cell apoptosis [44]. Baldari et al. [45] found that hyperglycemia could trigger the degradation of HIPK2 protein, consequently inhibiting p53-induced apoptosis and promoting tumor progression. But in the hypoglycemic environment, the degradation of HIPK2 can be attenuated. Lowering blood glucose level maintains the HIPK2/p53 apoptotic axis function. Studies have shown that chronic inflammation markers (like IL-6, TNF-α, COX-2) produced under hyperglycemic conditions can exert anti-apoptotic activity to cells and induce EMT [46, 47]. Proto-oncogenes and tumor suppressor genes in humans are mutually restricted in controlling cell growth, but when mutated or inactivated, these genes can lead to tumor progression. The effect of hyperglycemia on p53 and inflammatory factors is shown in Fig. 1.

### Effect of hyperglycemia on the resistance of tumor cells to chemotherapeutic drugs

Studies showed that [48–51] elevated blood glucose during chemotherapy increased the chemoresistance of tumor cells. Ma et al. [48] revealed that hyperglycemia attenuated the antiproliferative effect of 5-Fluorouracil (5-FU) on colon cancer cells. Patients with colorectal cancer accompanied by hyperglycemia need a higher dose of 5-FU and longer chemotherapy to adequately inhibit tumor cell growth. Zhao et al. [49] pointed out that hyperglycemia attenuated the chemosensitivity of gastric cancer cells to 5-FU. Hyperglycemia increases the expression of Nampt and Sirt1 in gastric cancer tissues and the expression of mutant p53 (compared with wild-type p53, the overexpression of mutant p53 in tumor cells is positively correlated with the high level expression of P-gp), resulting in the up-regulation of P-glycoprotein (P-gp) and down-regulation of Topoisomerase IIα (Topo-IIα). P-gp is a typical chemoresistance-resistant protein marker, and Topo-IIα a target marker for anticancer drugs. Up-regulation of P-gp and down-regulation of Topo-IIα mean that hyperglycemia leads to drug resistance in gastric cancer cells.

Biernacka et al. [50] reported that high glucose inhibited the apoptosis of prostate cancer cells induced by docetaxel, which may be related to the increased expression of IGFBP2. After IGFBP2 was silenced with small interfering RNA (siRNA), hyperglycemia no longer conferred tumor cells the resistance to chemotherapy drugs. This result was consistent with other studies on esophageal cancer and breast cancer cells [51]. IGFBP2 inactivates the tumor suppressor gene phosphatase and tensin homolog (PTEN) deleted on chromosome ten, leading to chemoresistance [52]. The expression level of IGFBP-2 is positively correlated with the progression of breast, prostate, lung, and colon cancer [51]. Zeng et al. [53] found that in the hyperglycemic environment, the sensitivity of breast cancer cells to chemotherapeutic drugs (such as 5-FU, doxorubicin or paclitaxel) might be related to fatty acid synthase (FAS), since inhibiting fatty acid synthase restored the sensitivity and accelerated the apoptosis of breast cancer cells. Therefore, strict control of glucose in cancer patients may enhance the effectiveness of chemotherapy.

Metformin is the most common hypoglycemic agent that exerts a hypoglycemic effect by reducing hepatic gluconeogenesis and increasing peripheral glucose utilization. A cohort study by Libby et al. [54] found that metformin use in patients with type 2 diabetes reduced the cancer-related overall mortality and mortality. Cancer was diagnosed among 7.3% of 4,085 metformin users compared with 11.6% of 4,085 comparators. After adjusting gender, age, BMI, smoking and other factors, a significantly reduced risk of cancer was found to be associated with metformin: 0.63 (0.53–0.75). Studies have shown that metformin has proliferation-inhibiting and apoptosis-promoting effects on tumor cells [55, 56]. There are a number of mechanisms by which metformin has been reported to act and these include: (1) LKB1-dependent mechanism activated protein kinase (AMPK-mTOR) to inhibit tumor cell proliferation [57, 58]; (2) Significant activation of AMPK in MDA-MB-231 cells on normal blood glucose level [55]. When metformin was used to treat prostate cancer cells, the resistance of prostate cancer cells to docetaxel was inhibited under hyperglycemic conditions, indicating that metformin can restore the sensitivity of prostate cancer cells to docetaxel through decreasing IGFBP-2 levels [59].

There are conflicting views. Lee et al. [60] reported that the risk of prostate cancer was reduced in patients with diabetes. Some metabolic and hormonal factors, including blood glucose and insulin, may involve. However, Betancourt et al. [61] showed the reduced risk of prostate cancer in patients with diabetes might be attributed to the decline in testosterone levels in patients with diabetes. However, Xu et al. [62] revealed that pre-existing high-risk factors such as hyperglycemia or obesity were associated with poor prognosis of prostate cancer; Li et al. [22] pointed out that hyperglycemia increased the expression of miR-301a in prostate cancer cells, thereby promoting G1/S cell cycle transition in vivo and accelerating cell proliferation; Biernacka et al. [50, 51] found that high glucose inhibited the efficacy of docetaxel-induced apoptosis in prostate cancer cells, which may be associated with hyperglycemia-mediated IGFBP2 overproduction. This is the controversy about the relationship between hyperglycemia and prostate cancer. Some researchers believe that patients with diabetes have a lower risk of prostate cancer. However, others believe that diabetes or hyperglycemia may promote the progression of prostate cancer through promoting the proliferation of tumor cells and inhibiting the apoptosis of tumor cells. The latter view is consistent with the impact of diabetes or hyperglycemia on other types of cancer (such as breast, liver, pancreatic, colorectal, bladder, endometrial cancer, etc.).

## Conclusion

In summary, hyperglycemia accelerates the progression of tumor through enhancing the proliferation, migration, and invasion of tumor cells. However, the underlying mechanisms vary and still require more in-depth studies.

## Data Availability

Not Applicable.

## Abbreviations

5-FU: 5-Fluorouracil
AKT: Protein Kinase B
AMPK: Adenosine monophosphate-activated protein kinase
ATP: Adenosine triphosphate
CD147: Cluster of differentiation 147
CDK: Cyclin-dependent kinase
COX-2: Cyclooxygenase-2
EGF: Epidermal growth factor
EGFR: Epidermal growth factor receptor
EMT: Epithelial-to-mesenchymal transition
ERK: Extracellular signal-regulated kinase
FAS: Fatty acid synthase
FDG-PET: Fluorodeoxyglucose positron emission tomography
Glut1: Glucose transporter 1
GTPases: Guanosine triphosphatases
HIF-1: Hypoxia-inducible factor-1
HIPK2: Homologous domain-interacting protein kinase-2
HO-1: Heme oxygenase-1
IGF-1: Insulin-like growth factor-1
IGF-1R: Insulin-like growth factor-1 receptor
IGFBP2: Insulin Like Growth Factor Binding Protein 2
IL-6: Interleukin-6
MMP: Matrix metalloproteinase
mTOR: mechanistic target of rapamycin
p38 MAPK: p38 mitogen-activated protein kinases
PEG-CAT: polyethylene glycol-conjugated catalase
P-gp: P-glycoprotein
PI3K: Phosphoinositide 3-kinase
PTEN: Phosphatase and tensin homolog deleted on chromosome ten
ROS: Reactive oxygen species
siRNA: Small interfering RNA
SOD2: Superoxide dismutase
STAT3: Signal transducer and activator of transcription 3
TGF-β1: Transforming growth factor-β1
TNF-α: Tumor necrosis factor-α
Topo-IIα: Topoisomerase IIα
uPA: urinary plasminogen activator
VEGF: Vascular endothelial growth factor
ZIP10: Zin transporter 10
ZIP6: Zin transporter 6
